# The economic side of social remittances: how money and ideas circulate between Paris, Dakar, and New York

**DOI:** 10.1186/s40878-016-0039-6

**Published:** 2016-11-03

**Authors:** Ilka Vari-Lavoisier

**Affiliations:** 1 University of Pennsylvania, CASI, 3600 Market Street, Philadelphia, PA 19104 USA; 2 Institut de Recherche pour le Developpement, DIAL, 4, rue d’Enghien, 75010 Paris, France

## Abstract

This paper shows how economic remittances undergird the circulation of social remittances between New York, Paris, and Dakar. It compares the transnational practices of Senegalese-born migrants living in France and in the United States during the 2012 Senegalese presidential campaign to demonstrate how economic and political transnational practices mutually reinforce each other.

This paper contributes to scholarship in three key ways: it confirms the benefits of combining qualitative and quantitative transnational data, collected from origin and destination countries. It offers a welcome geographic extension to a literature on social remittances from which Africa remains absent. It makes a significant theoretical contribution by connecting economic sociology and migration studies to illuminate the impact of migrants’ transnational practices.

## Introduction

On March 26th, 2012, Macky Sall was elected President of the Republic of Senegal. This 50-year-old geologist won with 65.8 % of the votes in Senegal, but he won by a much greater margin abroad with respectively 82.5 and 84 % of the votes cast in the United States and in France.^1^ The media underscored the determinant role of the diaspora in Macky Sall’s victory. His incumbent, the outgoing President Abdoulaye Wade, claimed that he lost because the Senegalese diaspora living in “Western countries campaigned against him”.^2^ To which extent did absent citizens influence this presidential election?

To analyze the involvement of Senegalese-born migrants living in France and in the United States during the 2012 Senegalese presidential campaign, our research team gathered (1) census data (*N* > 42,000 individuals), (2) survey data (*N* = 953), and (3) qualitative data collected from Senegal, the United States, and France.

This empirical evidence shows that migrants’ economic power increases their voice, and their ability to affect electoral outcomes in their home country. In spite of their divergent socioeconomic profiles, Senegalese-born individuals living on both sides of the Atlantic exhibit a *high propensity to engage in cross-border economic practices* (census data). Comparing the activities of migrants in the U.S. and in France further confirms that e*conomic and political transnational practices interrelate* (survey data). Finally, the paper elaborates on the *mechanisms* that link economic and political transnational practices (through the analysis of qualitative material). It shows that non-migrants’ dependence on expatriates’ financial support enhances migrants’ propensity to influence their kin.

This project thus brings together studies focused on economic remittances (Carling, 2014; Gubert, 2007), on the one hand, and studies of social remittances (Levitt, 1998; Levitt, 2001), on the other. Economic remittances (or economic transfers) are all the monies and goods migrants send, from their host to their home country (Gubert, 2007). While social remittances (or social transfers) are the ideas, practices, and know-hows that circulate along migratory paths (Levitt 1998; Levitt 2001). To date, the literature does not examine how economic and social transnational transfers interrelate (Boccagni & Decimo 2013; Lacroix, Levitt, & Vari-Lavoisier 2016; Vari-Lavoisier 2016). This project moves the scholarship forward by developing a comprehensive approach to the multiple, distinct, albeit interdependent forms of transnational practices deployed by migrants. It underlines how a theoretical approach drawn from economic sociology (Zelizer, 2011, 2014, Zelizer & Tilly, 2006) can encompass and advance research investigating the migration-development nexus.

## First section: an interdisciplinary literature review

This first section shows that while the economic determinants of social remittances and the sociopolitical dimensions of economic remittances are understudied, interrelations between financial and social transfers remain largely unexplored.

### The financial dimension of social remittances

Most “*debates about migration and development privilege[d] the economic at the expense of the social. … But economics is not the whole story*” (Levitt & Lamba-Nieves, 2011, p. 2): migrants contribute to the development of their countries of origin through non-monetary transfers as well. To describe how ideas, practices, and know-hows travel along migratory paths, Peggy Levitt coined the term *“social remittances”* (Levitt, 1998, 2001; Levitt & Lamba-Nieves, 2011; Levitt & Rajaram, 2013). This concept marked a turning point in the analysis of the migration-development nexus. As a result, scholarship makes claims about the powerful impact of social transfers across a variety of contexts (Kapur, 2010, 2014; Lafleur, 2013; Levitt & Rajaram, 2013; Piper, 2009; Sasse, 2013; Tabar, 2014).

A set of studies that effectively launched social remittances as a core concept focused on the impact of mobility on gender roles, intra-familial relations, and households’ structures (Fargues, 2010; Gardner, 1995; Rahman, 2013). Subsequent research has indicated that social remittances can “scale-up” to affect national changes (Levitt & Lamba-Nieves, 2011, pp. 16–17). Studies conducted by political scientists and development economists, focusing on political outcomes and notably on electoral results, support this hypothesis (see Rapoport, 2015 for a review of the literature). For instance, Li and McHale (2006) compiled an extensive database to analyze the links between high-skilled migration in 1990 and the quality of institutions in sending areas in 2000. They found that migration had a positive impact on political institutions^3^ but a negative impact on economic institutions (Li & McHale, 2006). In the same vein, Spilimbergo (2009) argues that foreign-educated individuals promote democracy back home. While Beine and Sekkat (2013) insist on the extent to which the characteristics of the destination countries mediate migrants’ influence on the sending states.

Studies focusing on one destination country put forth similar findings. For instance, Lisa Chauvet and Marion Mercier studied the links between migration, electoral participation, and political competition in Mali. Using a panel dataset combing the Malian censuses (of 1998 and 2009) with electoral results (for the 1999 and 2009 municipal elections), the authors found a positive impact of migrants coming back from non-African countries on indicators of electoral competitiveness. The authors thus confirm that the effects of mobility vary according to returnees’ country of destination (Chauvet & Mercier, 2014). However, they cannot control for the fact that migrants located in OECD countries are also more likely to benefit from more economic resources. Along the same lines, Batista and Vicente (2011) conducted a voting experiment to assess the impact of international emigration on the political institution of Cape Verde. They isolated a positive effect of emigration on the demand for political accountability. However, the authors do not account for the incidence of migrants’ economic transfers in spite of the fact that their dataset would allow them to do so. These studies globally ignore the role of monetary resources in the circulation of political preferences and practices.

This line of research has been almost exclusively explored by political scientists thus far. For instance Merino (2005) was among the first to argue that the receipt of remittances undermines clientelistic practices. In the same vein, Thomas Pfutze (2012; 2014) studies whether economic transfers affect political outcomes. He argues that international migration promotes the “*quality of democracy*” in the sending country. He puts forth a simple mechanism: economic remittances contribute to increases in households’ income that “*make clientelism unambiguously more costly and, therefore, reduce turnout for the party engaging in clienteslistic arrangements*” (Pfutze, 2014, p. 306). In this respect, Merino or Pfutze adopt an instrumental approach to voting behaviors, reducing the impact of migration to an income-effect that would weaken voters’ interest to engage in clientelistic relationships. This perspective ignores migrants’ propensity to carry different political views.

More broadly, studies conducted by migration scholars, economists, and political scientists (studying social remittances, social transfers, or transfers of political norms) share an important blind spot: they largely overlook the abundant literature on *economic* remittances. The literature on social transfers thus suffers from an unsatisfying conceptualization of the role of economic remittances, reducing these transfers to mere monetary transactions. Consequently, some authors oppose social and economic remittances, claiming that they would have contradictory effects (Meseguer, Lavezzolo & Aparicio, this issue). Although this opposition between social and economic remittances makes little sense. Indeed, this paper shows that *economic remittances play a critical role in maintaining asymmetric cross-border ties along which social remittances circulate*.

### From development economics to migration studies

Accordingly studies of economic remittances exclude the literature on social transfers. Although recent publications explore the *social dynamic* underpinning economic remittances (see Carling, 2014; Garip, Eskici, & Snyder, 2014; Lacroix 2014).

More specifically, I contend that recent contributions are moving towards a *relational* accounting of migrants’ remittances. In other words, monetary transfers are part of interpersonal, and sometimes, intimate relationships. Economic remittances are critical to sustain ties, both *between non-migrants and migrants* and *between migrants* at destination (Carling, 2014; Garip et al., 2014; Lacroix, 2013, 2014; Senne, Chort, & Gubert, 2011).

Carling provides a welcome synthesis of the manifold scattered ethnographic evidence on remittances and shows how remitting behaviors follow *scripts* recognized by given social groups. Remittance transfers “*need to be seen as compound transactions with material, emotional, and relational elements”,* Carling states (2014, p. 219). Based on an extensive review of ethnographic studies, this claim is consistent with the conclusions of quantitatively oriented researchers. Remitting is a social practice, “*not just an individuals’ choice*” (Page & Mercer, 2012).

Garip et al., (2014) also emphasize migrants’ social incentives to remit. They test the hypothesis that remittances stem from “*interrelated individual behaviors*”. Using a longitudinal dataset collected from Thailand in 1994 and 2000 (*N* = 6,301 households for a total of 35,122 individuals), the authors find that migrants are more likely to remit as the number of remitters in their household of origin increases, or the share of remitters in their community of origin increases. Remitting behaviors are fostered by shared social norms, the authors conclude. Along the same lines, an innovative multi-sited research design investigated the impact of international migration on West African sending states (Senne et al., 2011). Senne, Chort and Gubert pinpoint how networks, *at destination*, enforce remitting behaviors. Networks play a double role, providing migrants and their families with services and information, the authors posit. Migrants’ propensity to remit increases when they value more the services the network might provide (Senne et al., 2011, p. 23). Remitting behaviors are thus inseparable from migrants’ networks.

These empirical findings corroborate the theoretical framework unfolded by Lacroix (2013, 2014). The author extensively studied collective economic remittances across different contexts, ranging from Morocco and Algeria to Punjab. He shows that economic transfers can be seen as “*communicative acts* i.e. as *messages* or public stances migrants endorse regarding their obligation towards their origin community” (2014). Qualitative (Carling, 2014; Lacroix, 2014) and quantitative (Garip et al., 2014; Senne et al., 2011) approaches thus converge toward the idea that economic remittances are spurred by *relational dynamics* rather than economic concerns.

However, the focus on migrants’ *motives* to remit does not account for the *impact* of economic remittances *on relationships*. In this respect, economic sociology proposes a relevant framework to develop a more comprehensive approach to migrants’ transfers.

### Economic remittances as transnational relational work

With its objective to move beyond the *“hostile worlds”* view that opposes “the social” and “the economic” (Zelizer, 2005), economic sociology is prone to studying the non-economic dimensions of monetary transactions. Zelizer’s perspective is critical to account for the fact that when actors engage in a material transaction, they mobilize much more than their economic resources; as a result, their transactions convey material but also symbolic and emotional outcomes (Bandelj, 2009; Zelizer, 2012).

To date, the field of economic sociology largely ignores migrants’ economic remittances, as noted by Zelizer (2014, p. 14). Although, economic remittances represent perfectly the social nature of monetary transactions. As stated above, remittances are fostered by individuals’ relationships at origin (Garip et al. 2014), and at destination (Senne et al. 2011), they make sense in the context of a given social group (Carling 2008, 2014) and they contribute to reaffirm migrants’ belonging to their community of origin (Lacroix 2013, 2014). As such, economic remittances are a telling illustration of the concept of “relational work” coined by Viviana Zelizer (Zelizer, 2005; Zelizer, 2011). Through economic transactions, participants “*perform unceasing relational work. Far from a Monopoly game in which people deploy stylized cash in pursuit of their own individual advantage, we find participants investing effort in establishing, transforming, and sometimes terminating interpersonal relations*” (Zelizer, 2014, p6). This perspective is germane to the analysis of the interpersonal consequences of monetary transactions. Economic sociologists further show that “*to accomplish exchange, participants have to negotiate these economic-qua-social relationships. This activity […] has repercussions for economic and social equality or inequality between participants*” (Bandelj,^ 2012^, p. 190).

This project states that drawing upon theoretical approaches to economic sociology enable us to encompass disparate but compatible findings on social remittances on the one hand, and on economic remittances on the other. This paper thus makes a significant theoretical contribution by connecting economic sociology and migration studies to illuminate the impact of migrants’ transnational practices. It also confirms the benefits of combining qualitative and quantitative *transnational* data. Finally, it offers a welcome geographic extension to a literature on social remittances from which Africa remains largely absent.

## Second section: the Senegalese context

There is little empirical evidence to document the extent to which African migrants contribute to the circulation of ideas and practices from their host to their home countries, in spite of the predominant role of migrants in the economic and political life of the continent.

Yet, reports urging policy makers to leverage diaspora resources to improve development are manifold (Ratha, Mohapatra, Ozden et al., 2011). The potential impact of human mobility is considerable, given that African countries have over 30 million international migrants across the globe. Migrants’ remittances to Africa exceeded US $40 billion in 2010, providing a lifeline to the poor in many African countries (Ratha et al., 2011). Among the few countries accounting for a substantial share of remittances to Sub-Saharan Africa, Nigeria is the largest recipient ($20 billion), followed by Senegal ($1.4 billion), Kenya ($1.2 billion), South Africa ($1.0 billion), and Uganda ($0.7 billion, *see* Ratha et al., 2011). This paper focuses on the second largest recipient of official transfers in Africa: Senegal.

### A diasporic state, a diversification of destinations

The Senegalese state has evolved from an immigration country to an emigration country since the mid-seventies (Fall, Carretero, & Sarr, 2010). If, initially, Senegalese emigrants were directed towards neighboring West African countries, they have increasingly made their way to Europe (Dia, 2010). As the former metropolis of Senegal, France is the historical destination of Senegalese emigrants in Europe. Senegalese migration to France started to be numerically visible in the mid-sixties, as a result of the active recruitment of workers initiated by the French automobile industry (Pison, 1997; Robin & Richard, 2000).

The Oil crisis in 1974 brought new restrictions on workers immigration in France, and stricter immigration laws were passed through the 1980’s (Babou, 2008; Chen, 2013). As But, the figure of people leaving the country increased largely in the 1980’s notably due to increasing economic difficulties in Senegal (Bruzzone, Gueye, & Sarr, 2006). New destinations emerged since: Italy became a major destination in the 1980’s, followed by Spain in the 1990’s (Lessault & Flahaux, 2014, p. 62).

### Always further: Senegalese migration to the U.S.

The Senegalese started coming to the United States during the mid-eighties. The number of Senegalese immigrants who arrived in the United States had a higher growth after the devaluation of the CFA currency in Senegal in 1990 (Babou, 2008, p. 158; Chen, 2013, p. 87). A series of legal dispositions benefited these new immigrants. The Amnesty Law of 1986 permitted undocumented Senegalese immigrants to obtain legal status. Largely as a result of this amnesty, the number of Senegalese-born individuals in the U.S. census tripled from 801 in 1980 to 2,426 in 1990, reaching 10,215 individuals in the 2000 census (Beck, 2008; Chen, 2013) and 21,804 individuals in 2012^4^ (see below). The creation of the Diversity Immigrant Visa Program (also known as the Green Card Lottery) which provides visas to natives of countries deemed to have low rates of immigration to the U.S. also gave some momentum to Senegalese migration to the U.S. (Thomas, 2011).

A large number of Senegalese migrants in the U.S. were initially rural migrants with little or no formal education, also known as “modou-modous” (Diop, 2008; Kane, 2011). However the profile of Senegalese *émigrés* has diversified over time. Immigrants arriving in the U.S. since the nineties had more formal education; and a sizable number of those who arrived after the nineties were students pursuing higher education in American graduate or professional schools (Chen, 2013).

Today, little is known regarding Senegalese assimilation in the U.S., although African, along with Asians, have the fastest rates of immigration to the U.S. (Rumbaut, 2004). Given the social implications of these new trends, studies are needed to better understand the implications of contemporary African migration to the U.S. (Thomas, 2011, p. 5). In particular, while the transnational economic practices of Senegalese migrants who settle in France have been studied since the nineties (Beauchemin, Hamel, Simon, & Héran, 2015; Delville, 1994, 2011), the cross-border activities of those located in the U.S. remain to be explored.

### Dependence on migrants’ remittances

This line of research is even more important given that, over the last decades, the Senegalese economy has become increasingly dependent on migrants’ remittances. Official figures estimate that economic remittances account for 13 % of Senegalese GDP. These estimates do not account for the high proportion of transfers channeled by informal operators (Charbit & Chort, 2006)^5^ but give a sense of the importance of remittances to the domestic economy. Over the period 2010-2012, remittances in Senegal equaled, on average, 52 % of exports of goods and services (World Bank, 2013) and have become the principal source of external financing for the Senegalese economy, far exceeding Foreign Direct Investments, external borrowing, and official development assistance (Cisse, 2011). Senegalese migrants send money back home from all over the world, but OECD countries remain the first source of remittances (see Tables 9 and 10).^6^ Arguably, nowadays, “*the true driver of Senegal’s economy is migrants’ income”* (Dahou & Foucher, 2004).

### From the economic to the political role of Senegalese expatriates

Given the importance of remittances in Senegalese national economy, the topic of migration has emerged as an increasingly prominent topic in the political discourse (Fall et al., 2010; Gerdes, 2007). As shown by Kapur and McHale (2005) across the globe, “the lucre of remittances” has led politicians to court their diaspora (p. 154). While migration challenges territorial conception of citizenship and sovereignty, nation states increasingly implement institutional structures aiming at tapping the (economic) resources of their diaspora (see Gamlen, 2014; Kuznetsov, 2013; Pellerin & Mullings, 2013). In Senegal, this is perceptible through the creation of: The Ministry of Senegalese abroad in 2009; The “Senegalese Diaspora Foundation” in 2010; and the “Superior Council of Senegalese Abroad” in 2010 as well (see Ratha et al., 2011, p. 177). In this respect, Senegal illustrates the broader tendency of origin states to pursue development by establishing institutions promoting emigrants’ implication in national politics (Gamlen, 2014). This institutionalization of the role of “residents abroad who are economically, socially and politically engaged in their country of origin” (Østergaard-Nielsen, 2003, p. 762) prompted political parties to court migrants in various destinations.

### Political context of the 2012 presidential election

Senegal’s last elections illustrate the expansion of transnational political practices. Since 2000, the major electoral campaigns were organized not only in Senegal but also in major migrants’ countries of settlement, notably the United States and France: “Ten per cent of Senegal’s population live and work abroad, making them an important constituency for Senegalese politicians” (Salzbrunn, 2004).

**Table:** 

*Box: Some Context* Senegal is considered as one of the most stable political democracies in Africa and is hailed for its long history of participating in mediation and peacekeeping in the region (World Factbook, 2013). Since its Independence (on April 4^th^, 1960) the country has neither suffered any *coup d’Etat,* nor been under authoritarian rule. From 1960 to 1980, the country was ruled by Leopold Sédar Senghor under de facto one-party rule (Beck, 1997, pp. 8–10). When Léopold Sédar Senghor retired in 1980 he handed over power to Abdou Diouf. Successfully reelected in 1983, 1988, and 1993, Abdou Diouf lifted political restrictions and legalized several opposition political parties between 1980 and 2000 (Beck, 1997, pp. 14-18). After two defeats (in 1988 and 1993), Abdoulaye Wade^7^ won the 2000 Presidential election. The subsequent peaceful transfer of political power between parties was praised as a “real democratic progress” (Fall et al., 2010, p. 9), confirming the maturity of Senegalese democracy. Nonetheless, A. Wade’s approach has been doomed as “dynastic” (Mbow, 2008, p. 156), especially after his reelection in 2007 (Diop, 2010; Fall et al., 2010, p. 7; Mbow, 2008, p. 158). President Wade notably amended the Senegalese Constitution over a dozen times to weaken the opposition and increase the executive power (World Factbook, 2013). For instance, the Constitution has been amended to allow A. Wade to run for a third mandate in the 2012 Presidential election, despite the constitutional two-term limit (Chen, 2013). *Macky Sall President* Wade’s intention to run for a third term encouraged critics. Throughout 2011 and early 2012, those opposed to Wade’s candidacy organized demonstrations. Despite apprehension about escalating public unrest, the first round of the presidential election took place on February 2012 without incident. Macky Sall finished second in the first round, with 26.6 % to Abdoulaye Wade’s 34.8 %. However, more than a dozen other contenders had united behind Macky Sall, urging their followers to vote for the challenger. The second run was held on March 25, 2012 and Macky Sall won 66.8 % of the votes. Abdoulaye Wade quickly conceded his defeat.^8^	

Many observers underlined that the diaspora played a key role both in “*ensuring Macky Sall’s presidential victory and in consolidating Senegal’s democracy”* (Chen, 2013). The defeated president himself claimed that *“he did not win the first round of the presidential election because “the Occident campaigned against him”, designating in particular the U.S. and France.”* 9 As a matter of fact, part of the electoral campaign was organized in these two countries. Paris and New York were considered “*key battlegrounds*” in the last Senegalese presidential campaigns, because they are “*strong symbols of the winds of change*” (Salzbrunn, 2004, p. 6). The number of electors registered on voting lists abroad is low (about 3.4 % of the total, see Dedieu, Chauvet, Gubert, Mesplé-Somps, & Smith 2014) but the candidates expected to trigger an avalanche that would impact subsequent elections back in Senegal. Expatriates have become “*ambassadors and vote multipliers”* for Abdoulaye Wade in 2000 (Salzbrunn, 2004, p. 10) and more recently for Macky Sall in 2012.

Official results confirm this hypothesis: the newly elected president Macky Sall benefited from a large support among expatriates. In the first round, Abdoulaye Wade was in the lead in Senegal but only in second place in France and third place in the United States, winning less than 20 % of the ballots cast in these two countries.^10^ In the second round, A. Wade lost by an even greater margin abroad. In France, Macky Sall won with 84 % of the votes and in the U.S., he won with 82.5 % of the votes,^11^ compared to 65.8 % in Senegal (Dedieu et al., 2014, p. 70). In spite of these striking differences, to date, neither journalists nor academics can “*provide an explanation for why the diaspora vote significantly magnifies national tendencies*” (Dedieu et al., 2014).

This paper unravels the channels through which expatriates participate in the political life of their country through the analysis of three complementary sources of data.

## Third section: data and methods

This project combines (1) qualitative data, (2) survey data, and (3) census data.

### Qualitative data collected both at origin and at destination

The qualitative evidence analyzed in this paper was collected from France and Senegal, between 2008 and 2012. A first pilot (2009) served as a basis for a collective data collection (2011 – 2012).

**Table:** 

Box: A pilot (2009 – 2010) In September 2009, I started my fieldwork in a Hometown Association (HTA) created in 1979 by villagers from Diarama, a small town in the Senegal River Valley. I attended multiple meetings and events organized by the HTA, and conducted interviews with members involved in the HTA, starting with the leaders and extending to members of various levels of involvement, and their relatives. After 1 year of fieldwork in France, I wrote my master thesis on the French chapter of this hometown organization. In September 2010, I started my PhD (at the Ecole Normale Superieure de Paris). In order to pursue the ethnography of this organization, I moved to Senegal, dividing my time between Dakar and Diarama over the course of twenty two months. At the same time, I took an active part in the implementation of the TIMME project, which aimed at replicating this approach in order to collect qualitative data from other migrants’ organizations.	

The institute of Research for Development funded and coordinated the implementation of an original data collection: The TIMME^12^ project. Its research design combined quantitative and qualitative methods to collect “a body of data with greater reliability and more internal validity than could be achieved using either method alone” (Massey & Zenteno, 2000).

First, we analyzed a quantitative database to gain insights on the universe of hometown associations (HTA’s) created by and for Senegalese migrants living in France and in Italy. To do so, we used an innovative transnational quantitative database: the survey MIDDAS.^13^

**Table:** 

Box: The MIDDAS survey 300 Senegalese migrants in France and 302 Senegalese migrants in Italy were interviewed during 2009 using common sampling methodology and questionnaire (see Senne et al., 2011 Detailed information on migrants’ personal networks in France and Italy has been recorded together with data on remittances, savings, investment projects, and migrants’ individual characteristics. Senegalese migrants living in Europe answered a dozen of questions concerning their eventual participation to Hometown Associations (HTAs) in their host country.	

We drew upon this quantitative data to identify case studies of migrants’ organizations to be studied in depth. These structures were chosen for their representativeness, as we were convinced that the question of “societal significance” cannot be “completely divorced from *the question of “statistical significance””* (Fitzgerald, 2006, pp. 15-16).

Third, we identified the chapters of those organizations, in France, in Dakar, and in the villages of origin. We established two teams of ethnographers, one in France and one in Senegal. The fieldwork started in September 2011 with parallel inquiries in the Senegalese and French chapters of the selected migrants’ organizations.

After eight months, both French and Senegalese research teams met in Senegal (see picture). The last phase of fieldwork was realized by bi-national duos of ethnographers in Dakar and in the different villages of intervention of these HTAs. Finally, we analyzed these data jointly and this cross-analysis of data by Senegalese and French ethnographers significantly advanced our understanding of economic and social remittances (Gubert, Mesplé-Somps, Sané, & Vari-Lavoisier, 2016).

The importance of assessing the extent to which our qualitative results reflect larger patterns motivated the design of a second phase of the project: a transnational exit poll.

### A transnational exit poll (Paris – New York – Dakar): “Sondage sortie des urnes”

This quantitative survey was conducted by economists and sociologists who cooperated to develop interdisciplinary research designs adapted to our research questions on social transfers. This data collection was fully financed, designed, and implemented by the research center DIAL (Institute of Research for Development, Paris, France).^14^

This “Sondage sortie des urnes” (2012) consisted in a quantitative data collection: 923 Senegalese voters were interviewed on the day of Senegalese elections at the exit of polling stations. The first phase was realized on the day of the presidential elections (February 26, 2012) in the United States and in France (respectively 354 and 199 questionnaires were collected in Senegalese consulates of Paris and New York). The second phase was realized on the day of legislative elections (July 1^st^, 2012) in France and in Senegal (respectively 163 and 207 questionnaires were collected in Paris and Dakar). This paper compares the American sample (*N* = 199) with the French sample (*N* = 558); it makes use of the data collected in Senegal (*N* = 163) to conclude.

Four main sections questioned Senegalese voters on their socio-demographic characteristics, the intensity of their transnational connections, their political or associative engagement, and their social transfers. This design enables me to compare how profiles, trajectories, and political behaviors differ according to the destination country – as well as to study how these variations mediate migrants’ influence on their homeland.

**Table:** 

Three indicators aim at measuring migrants’ influence on their relatives: (1) migrants’ involvement in the 2012 presidential campaign^15^; (2) migrants’ propensity to give voting recommendations to their relatives in Senegal^16^; (3) non-migrants’ propensity to take migrants’ voting recommendations into account (see Table 14 for wording of the questions and the construction of the variables of interest). The independent variables are: basic sociodemographic characteristics (age, sex, citizenship); respondents’ educational attainment (diploma); respondents’ occupation, income, and length of stay in the destination country; and, finally, the intensity of migrants’ contact with their relatives at origin (see Table 14 for the construction of the variables of interest and Table 3 for descriptive statistics).	

This survey presents advantages, such as its transnational design, prone to compare the transnational practices of Senegalese-born migrants in two destination countries, and to confront migrants’ and non-migrants’ responses, notably regarding the influence exerted by migrants on their relative at origin. However this survey also presents serious limits: the limited sample size, on the one hand; and the lack of representativity of voters, on the other. The population of voters is likely to be non-randomly selected; this might induce a bias in our analysis of migrants’ political practices.

### Census American data and nationally representative French data on Senegalese-born population in each country

To put into perspective the results of this exit poll survey, I compare each subsample (collected in France and in the U.S.) with the general population of Senegalese-migrants located in both countries.

For the U.S., I analyze data compiled by the Census Bureau: the American Community Survey^17^ provides exhaustive data on the population of Senegalese-born migrants living in the United States (estimates for the period 2011-2013). The total sample comprises 312,861,723 foreign-born individuals officially registered in the U.S., including 21,804 Senegalese-born individuals.^18^ This dataset covers basic sociodemographic characteristics, as well as detailed information regarding housing conditions, earnings, and occupation.

For France, I analyze a survey conducted by two national statistical agencies (INED^19^ and INSEE^20^) in 2008. This survey closes the gap, largely acknowledged, in data availability on immigrants (of first and second generations) in France. It is based on a sample of 21,761 individuals aged from 18 to 60.^21^ This sample is representative of the whole population living in metropolitan France: the 2007 census was used as sampling frame for the TeO survey and the data were weighted to produce nationally representative estimates.^22^

The questionnaire covers a wide range of topics (education, employment, migration history, family structure, social relationships, etc.). In addition, the questionnaire includes a module on relationships with the origin country. The data thus contains a great variety of indicators of integration, assimilation, and transnational activities.

## Fourth section: from political to economic transnationalism

A comparative lens discloses that, despite different sociodemographic profiles, Senegalese migrants living in the U.S. and in France exhibit a similarly high propensity to engage in cross-border political and economic activities.

### Comparing the general profile of Senegalese-born individuals living in France and in the U.S.

#### The determinants of transnational political participation

The historical links between France and Senegal are still perceptible through the size of the Senegalese diaspora, three times more important in the former metropolis than in the U.S. (see Tables 1 and 2). Also Senegalese immigration to the U.S. is more selective than immigration to France (62.6 % of emigrants living in the U.S. have at least some college while only 45.2 % of those living in France attended college). It gives credence to the idea that France has become a less attractive destination, in particular for elites, due to the growing impression that Senegalese-born migrants tend to be “*déclassés*” (Gueye, 2001, p. 130) and are discriminated against when it comes to employment or housing (Beauchemin et al., 2015, pp. 383–412).

**Table 1:** General population and voters sample

	Census data	
	USA^a^	France^b^
Sex and age		
Total population (N)	21,804	71,119
Male (%)	60.1	57.3
Female (%)	39.9	42.7
Marital status		
Population 15 years and over (N)	21,182	61,925
Now married (inc. separated, %)	58	60
Widowed (%)	1.3	1.9
Divorced (%)	9.0	3.5
Never married (%)	29.7	34.6
Educational attainment		
Population 25 years and over (N)	18,519	60,882
Less than high school diploma (%)	16.9	23.0
High school graduate (includes equivalency, %)	20.5	31.8
Some college or associate’s degree (%)	29.3	13.9
Bachelor’s degree and above (%)	33.3	31.3
Years of arrival		
Population born outside the United States/France (N)	21,804	71,119
Entered 2010 or later for the US/2007 or later for France (%)	12.2	6.27
Entered 2000 to 2009/2000 to 2007 (%)	41.8	27.7
Entered before 2000 (%)	46.1	24.1
Entered 1990 to 2000 (%)		23.0
Entered before 1980 (%)		11.9
NR (%)		6.9
Employment status		
Population 16 years and over (N)	21,141	71,119
Employed (%)	69.7	57.7
Unemployed (%)	6.3	12.9
Not in labor force/unknown (%)	23.5	29.4

Sources: ^a^ Census bureau, American Community Survey (2013). ^b^ INED, Trajectoires et Origines (2008)

**Table 2:** Transnational political practices (descriptive statistics)

	USA^a^	France^a^	Pooled^a^
Sex and age			
Total population (N)	196	558	754
Male (%)	71.4	72.4	72.1
Female (%)	28.6	27.6	27.9
Marital status			
Population 15 years and over (N)	196	558	754
Now married (inc. separated, %)	66.3	60.8	0.6
Widowed (%)	1	1.4	1.3
Divorced (%)	6.5	4.6	5.1
Never married (%)	24.6	31.7	29.9
Educational attainment			
Population 25 years and over (N)	196	558	754
Less than high school diploma (%)	26.1	20.9	22.2
High school graduate (includes equivalency, %)	10.6	21.2	18.4
Some college or associate’s degree (%)	30.2	22.5	24.5
Bachelor’s degree and above (%)	33.2	35.5	34.9
Years of arrival			
Population born outside the United States/France (N)	196	558	754
Entered 2010 or later for the US/2007 or later for France (%)	27.6	18.0	20.5
Entered 2000 to 2009 / 2000 to 2007 (%)	16.6	28.3	25.3
Entered before 2000 (%)	34.2	19.4	23.3
Entered 1990 to 2000 (%)	9.5	14.4	13.2
Entered before 1980 (%)	0.5	12.8	9.6
NR (%)	11.6	7.0	8.2
Employment status			
Population 16 years and over (N)	196	558	754
Employed (%)	78.7	78.1	78.4
Unemployed (%)	14.3	8.2	11.2
Not in labor force/unknown (%)	7.0	14.7	10.8
Citizenship	%	%	%
Senegalese only	81,7	60,8	66,2
Dual citizenship	18,3	39,2	33,8
Main motive for migrating (%)			
Work	25	12,8	16
Looking for work	14,4	15,3	15
Health issues	0,5	0,6	0,6
School/professional training	27,1	42	38,1
Problems with your marriage	0,5	0,6	0,6
Following/joining family members	15,4	24,8	22,3
Looking for independence	3,2	1,3	1,8
Other reason	13,8	2,7	5,6
Transnational practices in two destination countries			
Frequency of phone calls			
No contact	4,5	3	3,4
Daily	16,6	22,5	20,9
Weekly	41,2	49,6	47,4
Monthly	1	19,6	14,7
NR	36,7	5,3	13,6
Frequency of visits			
Never	16,6	9,3	11,2
At least once a year	5,1	11,4	9,7
At least once in the past	33,7	70,2	60,7
NR	44,7	9,1	18,4
Send economic remittances			
No	20,6	19,6	19,9
Yes	79,4	80,4	80,1
Own land/house in Senegal	60,3	51	53,4
Own a business in Senegal	15,1	11,8	12,6
Plan to return to live in Senegal	74,9	73,4	73,8
Vote in the country of destination if double citizenship (N= 256)	75	71,4	71,9
Vote in Senegal (N= 754)	58.3	40	42.6
Vote at destination - for dual citizenship holders	No	Yes	Total
No	40,1	11,9	28,1
Yes	59,9	88,1	71,9
N (Obs.)	196	558	754

Source: Institut de Recherche pour le Développement, DIAL, “Sondage Sorties des Urnes” (SSU, 2012). ^a^ Institut de Recherche pour le Développement, DIAL, “Sondage Sorties des Urnes” (2012)

As a matter of fact, “*African migrants in general”* are underrepresented amongst French *“political or economic decision-makers”* (Salzbrunn, 2004, p. 471). This can be especially frustrating for a population prone to engaging in political activities. Sub-Saharan migrants are indeed eager to participate in the political life of their country of destination when they are given the opportunity to do so. The TeO dataset (cf*. supra*) allows us to compare the electoral participation of individuals born in more than seventy countries, who are living in France today. The results indicate among foreign-born individuals who are entitled to vote (possessing the French nationality), migrants coming from Sub-Saharan Africa are more inclined to participate in French elections (amongst foreign-born individuals entitled to vote, 85.6 % of migrants coming from West Africa voted at least once in France, compared to 58.7 % of individuals originating from other European countries, see Table 3).

**Table 3:** Political participation at destination by place of birth (TeO)

	Vote in France		
Place of Birth	No (%)	Yes (%)	Total (N)
France	26.6	73.4	752,154
North Africa	34.3	65.7	161,480
Western Africa	14.4	85.6	8,529
Eastern Africa	29.6	70.4	32,738
Asia	37.2	62.8	26,790
European Union	24.9	75.1	30,505
Other European countries	41.3	58.7	26,324
Americas, Oceania	25.8	74.2	32,550
Total (N)	303,174	767,899	1,071,073

Source: INED, Trajectoires et Origines (2008). Weighted estimates. Sample: foreign-born migrants (1st generation) possessing the French nationality

Migrants who vote in their destination country are also more likely to vote in their country of origin: 33.8 % of migrants who voted at destination also voted in their home country, while 30.8 % of those who did not vote in France participated in elections abroad (respectively 78.1 % and 70.6 % if we restrict the analysis to individuals possessing the French nationality, see Table 4).

**Table 4:** Cumulative political participation?

All foreign-born migrants	Vote in the country of origin		
Vote in the country of destination	No (%)	Yes (%)	Total (%)
	%	%	%
No (%)	69.2	66.2	68.7
Yes (%)	30.8	33.8	31.3
Total (N)	2,259,040	457,587	2,716,627
Migrants with French citizenship	Vote in the country of origin		
Vote in the country of destination	No (%)	Yes (%)	Total (%)
No (%)	29.4	21.9	28.3
Yes (%)	70.6	78.1	71.7
Total (N)	910,321	160,752	1,071,073

*Source*: INED, Trajectoires et Origines (2008). Weighted estimates. Sample: foreign-born migrants aged 18 to 50

This “cumulative participation” suggests that “*electoral mobilisation in host countries helped to consolidate electoral mobilisation in countries of origin*”, advances Dedieu et al. (2014) who recently looked at Senegalese political practices. Exploring the determinants of transnational voting behaviors, the authors show that, compared to the general population of Senegalese migrants, Senegalese voters tend to be better-educated and more integrated at destination. For instance, almost half of the individuals who voted at destination (47 %) had attended college, while this group only represents a quarter of the total reference population of Senegalese migrants (24 %). This contribution underlines that transnational voting behaviors are correlated with the same “heavy” socioeconomic variables than national electoral participation: education, employment, and income (Dedieu et al., 2014, p. 67). These findings are in line with previous studies underscoring that migrants’ propensity to engage in transnational activities is a function of their assimilation at destination (Guarnizo, Portes, & Haller, 2003; Itzigsohn & Saucedo, 2002; Levitt, 1998; Portes, Guarnizo, & Haller 2002; Portes & Zhou, 2012).

To date, most studies relate transnational practices to migrants’ trajectories at destination or to the characteristics of their community at destination (see Martiniello & Lafleur, 2008 for a review of the literature). This paper expands upon these findings by showing that political participation is irreducible to socioeconomic determinants. We need to factor in individuals’ *agency*. Migrants’ transnationalism also reflects their personal projects, and notably their intentions to return to their homeland in the medium or long run.

### Who are these transnational activists? The temporal dimension of transnational practices

Migration scholars largely explored the spatial dynamics of migrants’ transnationalism; *“in contrast, little is said about the temporality of transnational practices*” (Lacroix, 2015, p. 11). This is all the more surprising that migrants’ long term plans (and notably their intentions to return) shape individuals’ trajectories at destination and their potential contribution to the development of their home countries (Flahaux, 2015). In an effort to account for the temporal dimension of migrants’ engagement, I use the TeO dataset (see Third section: data and methods) to compare the transnational practices of migrants coming from more than the five continents. I assess the extent to which individuals’ projects to either stay in their country of destination or return to their homeland shed light on their transnational commitment.

Multiple indicators confirm that migrants who are planning on going back are more prone to engage in transnational political practices (see Table 5). The latter are more interested in the political life of their country of origin (16.9 % are really interested in the political life of their home country compared to 9.7 % of those who are not planning on going back). They are also more likely to participate in the elections of their homeland (16.4 % vote in their home country, compared to 15.2 % for those who are not planning on returning, respectively 24.5 and 14.4 % among Senegalese migrants).^23^

**Table 5:** Plans to return and transnational political practices

Interest in the politics of the country of origin	Plan to return			
All foreign-born migrants	Plan to return: No (%)	Yes (%)	Maybe (%)	Total (%)
Very interested	9.7	16.9	18.3	15.5
Somewhat interested	16	20.8	23	20.1
Not too interested	32.4	31.2	32.5	31.7
Not at all interested	41.8	30.6	26.2	32.4
Total (N)	634,799	1,593,284	488,543	2,716,627
Senegalese-born migrants	Plan to return: No (%)	Yes (%)	Maybe (%)	Total (%)
Very interested	25.1	26.4	21.9	25.6
Somewhat interested	13	19.6	31.6	20.8
Not too interested	36.8	32.5	37.8	33.7
Not at all interested	25.1	21.6	8.7	19.9
Total (N)	5,526	41,814	8,660	56,001
Vote in the country of origin	Plan to return			
All foreign-born migrants	Plan to return: No (%)	Yes (%)	Maybe (%)	Total (%)
No (%)	84.8	83.6	79.7	83.2
Yes (%)	15.2	16.4	20.3	16.8
Total (N)	634,799	1,593,284	488,54	2,716,627
Senegalese migrants	Plan to return: No (%)	Yes (%)	Maybe (%)	Total (%)
No (%)	85.6	75.5	70.6	75.7
Yes (%)	14.4	24.5	29.4	24.3
Total (N)	5,526	41,814	8,660	56,001

Source: INED, Trajectoires et Origines (2008). Weighted estimates. Sample: foreign-born migrants aged 18 to 50

This suggests that transnational political activities cannot be seen as a mere consequence of migrants’ present socioeconomic positions, but relate to migrants’ long term plans. Migrants’ transnational practices are geared towards the preparation of a better future for their kin and for themselves. This perspective simultaneously illuminates why political and economic transnationalism are interrelated, as the next section shows.

### The economic underpinning of migrants’ political transnationalism

Various forms of transnational practices have garnered increased attention from scholars since the 1990’s but researchers usually arranged these activities according to the typology of economic, political, and sociocultural transnational practices (see Beauchemin, Lagrange, & Safi, 2011; Itzigsohn & Saucedo, 2002; Portes, Guarnizo, & Landolt, 1999). Such classifications stimulated useful analytical distinctions, but simultaneously concealed to what extent these practices are mutually constitutive.

The empirical evidence analyzed in this paper demonstrates the strong interdependencies between political involvement, economic investments, and plans to return to the homeland. The Tables 6 and 7 confirms that migrants who remit are more likely to participate in the elections of their country of origin – a tendency which is stronger among West African migrants in general, and Senegalese nationals in particular.

**Table 6:** Interrelations between economic and political transnationalism

	Interest in the politics of the country of origin	Very interested	Somewhat interested	Not too interested	Not at all interested	Total (N)
All foreign-born migrants						
Remit	No (%)	1.7	3.1	5	8.3	22,533,078
	Yes (%)	17.1	14.2	22.1	18.7	580,516
	Total (%)	2.1	3.3	5.5	8.6	23,113,594
Collective Investments	No (%)	9.9	16.1	27.8	45.1	4,244,844
	Yes (%)	22.3	27.5	26.3	23.6	310,133
	Total (%)	2.1	3.3	5.5	8.6	23,113,594
Own a house in the country of origin	No (%)	1.7	3	4.9	8.3	22,477,853
	Yes (%)	15.5	16.7	24.4	20.7	635,741
	Total (%)	2.1	3.3	5.5	8.6	23,113,594
Economic investment in the country of origin	No (%)	2.1	3.3	5.4	8.6	23,034,338
	Yes (%)	10.9	18	14	9.1	79,256
	Total (%)	2.1	3.3	5.5	8.6	23,113,594
Senegalese migrants						
Remit	No (%)	21.3	21.6	31.7	25.4	35,083
	Yes (%)	34.4	20.4	33.5	11.7	24,547
	Total (%)	26.7	21.1	32.4	19.8	59,630
Collective Investments	No (%)	26.9	20	32.6	20.5	47,155
	Yes (%)	26	25.3	31.5	17.3	12,474
	Total (%)	26.7	21.1	32.4	19.8	59,630
Own a house in the country of origin	No (%)	27.1	21.6	32.8	18.5	46,123
	Yes (%)	25.6	19.1	31.1	24.3	13,506
	Total (%)	26.7	21.1	32.4	19.8	59,630
Economic investment in the country of origin	No (%)	27.4	20.4	32	20.1	57,846
	Yes (%)	4.8	42	44	9.1	1,783
	Total (%)	26.7	21.1	32.4	19.8	59,630

**Table 7:** Interrelations between economic transnationalism and voting behaviors

	All foreign-born migrants		Senegalese migrants	
Vote in the country of origin	No (%)	Yes (%)	No (%)	Yes (%)
Remit (yes, %)	1.9	11.5	19.3	30.4
Collective Investments (yes, %)	9.8	25.5	17.7	47.5
Own a house in the country of origin (yes, %)	1.8	15.8	20.7	34.9
Economic investment in the country of origin (yes, %)	2.1	19.6	23.7	30.6

Among Senegalese-born individuals, 19.3 % of those who do not remit participate in elections in Senegal, this proportion reaching 30.4 % among those who remit. Similarly, 20.7 % of respondents who do not own a house in Senegal vote in Senegalese elections; this proportion reaching 34.9 % among those who own a house. Multivariate analyses confirm these correlations between political participation at origin and remitting behaviors (see Table 8).

**Table 8:** Electoral participation at origin

	Vote in the country of origin		
	Model 1	Model 2	Model 3
Age (years)	0.00591	0.00852*	0.00983*
	-1.35	-2.05	-2.35
Years since arrival	0.00765	0.00308	0.00448
	-1.92	-0.8	-1.16
Sex: female	-0.0645	-0.0884	-0.102
(ref: male)	(-1.03)	(-1.43)	(-1.62)
Laborers	-0.146	-0.138	-0.121
	(-0.81)	(-0.72)	(-0.64)
Executive and managers	0.253	0.261	0.276*
	-1.9	-1.96	-2
Inactive	0.135	0.117	0.0944
	-0.69	-0.58	-0.45
Income/head	-0.000101 -	0.0000795	-0.0000882
	(-1.95)	(-1.55)	(-1.69)
Remit: yes	0.162**		
(ref: no)	-2.85		
Collective investment: yes		0.168**	
(ref: no)		-2.71	
Economic investment			0.214
In the country of origin: yes			-1.43
N (obs)	238	238	238

Note: Table reports marginal effects of the logit specification. For binary variables, the marginal effects are the differences between the two categories of the independent variable. T-statistics in parentheses (*p<0.1 **p<0.05, ***p<0.01). Sample: Senegalese-born migrants. Source: TeO survey (INSEE/INED, 2008)

The second dataset (SSU survey data, see Third section: data and methods) allows me to further test the interrelations between economic and political practices, on a smaller sample but with a broader set of indicators of political participation. Table 9 displays the significant and positive correlation between owning a house (or land) in Senegal and involvement in the electoral campaign.

**Table 9:** Participation in the Senegalese Electoral Campaign from the US/France

Campaigned in 2012 (marginal effects)	1	2	3	4	5
Age (years)	-0.006***	-0.005***	-0.004***	-0.0046**	-0.0052*
	(-5.97)	(-3.50)	(-3.41)	(-2.61)	(-2.44)
Sex (female)	-0.0549***	-0.0565***	-0.0490***	-0.0381***	-0.0418*
	(-5.48)	(-10.38)	(-54.56)	(-7.89)	(-2.09)
Length of stay abroad	0.00225	0.00330	0.00336	0.00212	0.00328
(years)	(0.88)	(1.51)	(1.62)	(0.88)	(1.50)
Employed	-0.126*				
(ref. unemployed)	(-2.07)				
Own land in Senegal	0.104***	0.114***	0.0922***	0.0762***	0.0453*
(ref: no)	(6.82)	(5.60)	(4.09)	(5.07)	(2.48)
Own a business in			0.0727	0.0644	0.0938*
Senegal (ref: no)			(1.88)	(1.76)	(1.98)
Member of a political				0.142***	0.0388
party at destination				(3.35)	(1.44)
Member of a Senegalese					0.317***
political party: yes					(13.69)
Education: High School		-0.0170	-0.0153	-0.0384	-0.0440
(ref. none)		(-0.20)	(-0.19)	(-0.48)	(-0.61)
Some College		-0.0135	-0.0165	-0.0324	-0.0198
		(-0.14)	(-0.18)	(-0.34)	(-0.24)
Bachelor		0.0795	0.0723	0.0454	0.0566
		(0.72)	(0.69)	(0.47)	(0.70)
Send economic			0.0344	0.0192	-0.0112
Remittances: yes			(0.86)	(0.58)	(-0.51)
Income: $16,000 to		0.0613***	0.0475***	0.0531***	0.0507**
$40,000 (ref: <$16,000)		(3.78)	(4.48)	(3.44)	(2.92)
Income: over $40,000		-0.0477	-0.0613	-0.0544	-0.0445
		(-0.81)	(-0.96)	(-0.96)	(-0.76)
Plan to return to Senegal			0.0453	0.0467	0.0316
(ref: no)			(1.48)	(1.67)	(1.01)
N	754	754	754	754	754

Note: Table reports marginal effects of the logit specification. For binary variables, the marginal effects are the differences between the two categories of the independent variable. T-statistics in parentheses (*p<0.1 **p<0.05, ***p<0.01). Source: SSU survey (DIAL, IRD 2012)

Table 10 displays the significant and positive correlation between owning a house (or land) in Senegal and the probability of being a member of a Senegalese political party. We can also note the robust correlation between sending economic remittances and being a member of a Senegalese political party. Migrants who were the most active in the 2012 electoral campaign maintain not only political but also *economic* ties with Senegal. Migrants’ political engagement is spurred by their *“strong economic interests”* back home (Kapur, 2014, p. 484).

**Table 10:** Membership of a political party in Senegal (migrants living in the US/France)

Member of a political party (marginal effects)	1	2	3	4	5	6
Age (years)	0.00367	0.00293	0.00158	0.000737	-0.000235	0.000351
	(1.68)	(1.35)	(0.65)	(0.41)	(-0.12)	(0.15)
Sex (female)	-0.0279	-0.0252	-0.00451	-0.0197	-0.000510	0.000573
	(-0.54)	(-0.46)	(-0.09)	(-0.38)	(-0.01)	(0.01)
Employed	0.101	0.135**	0.141***			0.131***
(ref. unemployed)	(1.87)	(2.74)	(3.69)			(5.57)
Income: more than $16,000	0.102***	0.0966***	0.0865***	0.00432	-0.00350	
(ref: <$16,000)	(7.07)	(4.69)	(10.97)	(0.14)	(-0.12)	
Income: over $40,000	0.0683**	0.0564**	0.0426	-0.0286	-0.0427*	
(ref: <$16,000)	(2.93)	(2.69)	(1.76)	(-1.65)	(-2.19)	
Plan to return to Senegal	0.0898*		0.0385	0.0593	0.0416	0.0389
(ref: no)	(2.35)		(0.70)	(1.18)	(0.77)	(0.81)
Send economic remittances		0.174*	0.141*	0.129*	0.113*	0.140*
(ref: no)		(2.44)	(1.99)	(2.39)	(2.55)	(2.34)
Own land/house in Senegal			0.117***		0.113***	0.116***
(ref: no)			(5.32)		(3.99)	(4.39)
Length of stay abroad (years)				0.000699	-0.000198	0.000000385
				(0.58)	(-0.16)	(0.00)
Education: High School				0.0492*	0.0446	
(ref. none)				(2.09)	(1.85)	
Some College				0.00903	0.00744	
				(0.55)	(0.44)	
Bachelor				0.00615	0.0110	
				(0.11)	(0.17)	
N	754	754	754	754	754	754

Note: Table reports average marginal effects of the logit specification. The marginal effects are evaluated at the means of the data. For binary variables, the marginal effects are the differences between the two categories of the independent variable. All regressions include country-fixed effects. T-statistics in parentheses (*p<0.1 ** *p*<0.05, *** *p*<0.01). Source: SSU survey (DIAL, IRD 2012)

One might argue that these correlations are driven by the fact that a more active political participation reflects a better socioeconomic status. Indeed, better-educated migrants come from privileged socioeconomic backgrounds. As such, they would be simultaneously more likely to own land or property in Senegal and to be engaged in political activities. Although, even after controlling for educational attainment, occupation at destination, income, and a series of other potential confounders, economic ties remain associated with a higher level of political transnationalism, *all other things held equal*. These results are consistent with the claim that the current literature overestimates the importance of educational attainment prior to migration and overlooks the incidence of current ties with their homeland, as well as individuals’ plans for the future.

Both political and economic ties hint at migrants’ projects of return. Transnational activists *invest* in their homeland – reminding us the polysemy of the term *investment* that refers both to the act of “investing money for profit or material result” and to the “act of devoting time, effort, or energy to a particular undertaking with the *expectation of a worthwhile result*” in the future.^24^ Taking into account individuals’ expectations sheds light on migrants’ incentives to dedicate resources to a country they left only temporarily. This approach paves the way for a more comprehensive understanding of the multiple and interrelated forms of transnationalism that have been studied in isolation thus far.

### Economic ties are multidimensional

Indeed, albeit classified as “economic”, practices such as owning a house or sending money to Senegal are not merely financial. Economic remittances relate to a series of other cross-border activities, usually considered as “social”, such as calling relatives back home or visiting them (see also Vickstrom, this issue). Economic remittances have a symbolic and communicative function. These transfers “*are to be understood as a communicative act,* i.e. *a mode of expression of emigrants through which they formulate what they think they have to do in accordance with who they think they are”* (Lacroix, 2010). Through their transfers, migrants *“take a stand on their plural being”* and remittances constitute a tangible mean through which migrants weave together the *“disjointed poles of their existence”*, here and there (Lacroix, 2014).

Peter Kankonde makes a similar argument when he underlines *“what lies beneath the surface of sent legal tenders is in fact the social and cultural subjective self of remitters as human beings and family members […] migrants remit primarily in a bid to foster familial belonging in order to escape social death and at the same time buy and sustain social status*” (Kankonde, 2010, pp. 225-240). Expanding upon these insights, I claim that migration scholars could benefit from recent findings emerging from the field of economic sociology in order to systematically study how economic transactions fulfill social functions.

### Economic sociology of migrants’ remittances

Economic sociology offers a conceptual framework especially relevant to advance our understanding of economic remittances, and to better comprehend the extent to which these transnational transactions are not exceptions standing alone in the socioeconomic life. To date, studies of migrants’ remittances constitute a subfield of migration studies that neither draw upon nor contribute to other fields interested in the social underpinning of economic life. This project suggests the heuristic decompartmentalizing of remittance studies.

The concept of *relational work* is especially relevant to describe how financial transactions (such as economic remittances) are irreducible to their monetary dimension. In “*all economic action*” Zelizer argues, “*people engage in the process of differentiating meaningful social relations*”. The process of relational work leads people to negotiate the exact content and nature of their relationships, notably through their material transactions (Zelizer, 2012, p. 146). Economic exchanges are integral to individuals’ constant efforts to create social boundaries and elaborate meaningful categories *“distinguishing persons, activities, and relations on different sides of the boundaries”* (Zelizer & Tilly, 2006, p. 17). Economic transactions are shaped, not only by economic objectives, but by interpersonal concerns as well.

Household economy offers a powerful illustration of the relational function of economic transactions. The circulation of money fulfills important symbolic and relational roles within families. As Zelizer notes: *“the mere establishment of a shared ancestry”* does not loom large without *“the actual performance of kinship”* (Zelizer & Tilly, 2006, p. 21). Money is vital to *perform* kinship, especially in West Africa (Boltz-Laemmel, 2013; Olivier de Sardan, 1996; Platteau, 2014) and this feature becomes blatant in transnational families. In the case of Senegalese migrants, remitting can be an explicit condition to maintain contact and retain ties, to remain a member of the family of origin and stay connected with friends and kin, as the case of Mariam shows.

My interview with Mariam takes place in rue Clerc, in the wealthy seventh arrondissement of Paris. The Eiffel Tower is two blocks away. Mariam works here, but she lives in La Courneuve, an hour away. She grew up in Saint-Louis, in Northern Senegal (3081 miles away) where she lived until 2005. At 22, she worked as a waitress in a small restaurant, where she met Mario, an Italian real estate agent. He told her about Europe and dreamt about living together. They left Saint Louis together, drove to Dakar, flew to Tunis and drove to Bizerte (Tunisia) where Mario had a small boat waiting for them. As they sailed to Naples, she became undocumented. They reached the Italian coast at night and settled in Florence. But after a violent argument, Mariam fled, took several trains to Lyon (France) and finally ended up in Paris, in 2007. In 2008, Mariam managed to become a legal resident in France and was eager to “testify” in her own words:

*“…write, write what I say ‘cause you know, they don’t know, they don’t realize (…) my family, you know, they believe I am a liar when I say that I have no money. You know, they think that migrants who don’t send [remittances], it’s because they are selfish. While… [she sighs] it’s so hard here. So hard, I never thought that it would be this way… (…) I know that life is hard for them [in Senegal] too. I know that but what can I do… I just can’t [help them]. See! See how I live, how hard I work… I just can’t (…) it’s tough but now, when I see a number starting with 212 [Senegal’s area code], I don’t pick up [the phone]… ‘cause I know that it’s for money, I don’t pick up ‘cause I know that they’ll ask for money… I understand them, I know that life is hard in Saint Louis (…) but what can I do? I have to pay my rent (…) If I work honestly, I barely make ends meet, I just can’t send [remittances]… (…) I’m really sad about that, sure, ‘cause… because of that I don’t have news anymore, I don’t know how they are doing (…),I’m often worried… If something happened to my parents, or my brothers, I would even not know about it. (…) Tho now that I’m here, I try to make my way… even if it’s really not like what that I imagined my life to be…”*

Interview with Mariam B.,

Paris (VII° arrondissement), April 2009.

Mariam’s inability to send money to her relatives at origin led to the termination of her relationships with her parents, siblings, friends and relatives in Senegal. Economic remittances illustrate that economic activity and intimate relations do not constitute separate spheres: *“people constantly mingle economic activity with intimacy”* (Zelizer, 2012, p. 152). In the case of transnational households, economic remittances signal the migrant is still part of the household; that is why no hardship and no misery in the host country can justify not sending money. The urge to maintain relationships leads migrants to transfer money in spite of sometimes truly difficult conditions. It has led scholars to show that remittances can flirt with the *“sacrifice”* (Fouron & Schiller, 2001; McKenzie & Menjívar, 2011; Singh, 2013). These patterns also account for the fact that economic transfers are typically sent in relatively small magnitudes at high frequencies (Yang, 2011) due to their function in fostering contacts and being part of the household’s daily life in spite of distance. It illuminates the correlations between phone calls, visits, and financial remittances: they are intrinsically linked (see Table 11).

**Table 11:** The relational role of economic remittances

Send economic remittances (marginal effects)	1	2	3	4	5	6
Age (years)	0.00163*	0.00242	0.00217	0.00240*	0.00265**	0.00331***
	(2.43)	(1.90)	(1.83)	(2.03)	(2.70)	(5.55)
Sex (female)	-0.0590	-0.0637	-0.0608	-0.0537	-0.0551	-0.0409
	(-1.18)	(-1.16)	(-1.18)	(-1.38)	(-1.47)	(-1.09)
Length of stay abroad	0.00160	-0.000199	-0.000181	0.000312	0.000538	0.000335
(years)	(1.08)	(-0.32)	(-0.25)	(0.89)	(0.65)	(0.29)
Employed	0.160***	0.151***	0.149***			
(ref. unemployed)	(7.20)	(9.30)	(8.44)			
Freq. of phone calls: daily	0.104	0.0946	0.0562	0.0701	0.0729	0.0458
(ref: every three months)	(1.89)	(1.69)	(1.08)	(1.31)	(1.29)	(1.18)
Freq. of phone calls: weekly	0.137**	0.135**	0.105*	0.109**	0.110*	0.0746*
	(3.22)	(2.79)	(2.41)	(2.72)	(2.47)	(2.57)
Freq. of phone calls: monthly	0.0574	0.0623	0.0396	0.0468	0.0487	0.0252
	(1.41)	(1.49)	(0.94)	(1.01)	(0.98)	(0.63)
Freq. of phone calls: none	-0.0747	0.00271	-0.0188	-0.0215	-0.0177	-0.0260
	(-1.25)	(0.04)	(-0.33)	(-0.33)	(-0.24)	(-0.38)
Freq. Of visits to Senegal:		0.141***	0.150***	0.153***	0.148***	0.149***
more than once a year		(3.92)	(3.72)	(6.76)	(3.50)	(4.37)
(ref: every 2 - 3 years)						
Less than once a year		0.126*	0.129*	0.116	0.114	0.107
		(2.20)	(2.20)	(1.85)	(1.62)	(1.67)
Never		0.0128	0.0195	0.0261	0.0228	0.0269
		(0.28)	(0.39)	(0.50)	(0.40)	(0.62)
Member of a political party in Senegal			0.0809*	0.0700*	0.0699*	
			(2.28)	(2.36)	(2.43)	
Income: $16,000 to $40,000				0.167*	0.167*	0.150
(ref: <$16,000)				(2.01)	(2.01)	(1.74)
Income: over $40,000				0.194***	0.189**	0.179**
(ref: <$16,000)				(5.02)	(3.11)	(3.09)
Education: High School					0.0000866	-0.0130
(ref. none)					(0.00)	(-0.28)
Some College					0.0270	0.0113
					(0.59)	(0.24)
Bachelor					0.0213	-0.00220
					(0.23)	(-0.03)
Member of a political party at destination:						0.0540*
Yes (ref: no)						(2.02)
Plan to return to Senegal: yes						0.129***
(ref: no)						(3.49)
	754	754	754	754	754	754

Note: Table reports average marginal effects of the logit specification. The marginal effects are evaluated at the means of the data. For binary variables, the marginal effects are the differences between the two categories of the independent variable. All regressions include country-fixed effects. T-statistics in parentheses (*p<0.1 **p<0.05, ***p<0.01). Source: SSU survey (DIAL, IRD 2012)

These findings are consistent with an abundant literature claiming that economic remittances reaffirm migrants’ belonging to a household located thousands of kilometers away (Carling, 2008; Kankonde, 2010; Lacroix, 2010; Van Hear, Vertovec, & Pieke 2004). In this respect, taking into account the *relational* nature of economic transactions enables me to move the scholarship forward and describe how *economic remittances participate in maintaining the transnational ties along which social remittances circulate*.

### How economic remittances foster social remittances

#### Economic remittances favor migrants’ influence

A relational perspective shows that economic transactions contribute to the renegotiations of the respective positions of the stakeholders. The concept of relational work underscores the efforts by individuals to negotiate constantly the content, the meaning of the relation, and the *reciprocal positions* of the partners (Tilly, 2006, p. 15). Relational work consists of sustained reciprocal efforts between the partners.”*But reciprocity doesn’t mean equality. Just think of the Hegelian conundrum of the master and slave”* (Bandelj, 2012, p. 180). Undeniably, if financial remittances signal the maintenance of transnational ties, they reinforce asymmetrical ties between senders and receivers. Elements of “*power are integral to any relational work”*, Bandelj adds (Bandelj, 2012, p. 180). In other words, economic remittances reinforce asymmetrical relations and these asymmetries pave the way for the circulation of social remittances. The acceptance of social remittances also reflects the domination of one actor on the other (see Levitt, 2001).

To test this hypothesis, I use the survey data (see Third section: data and methods) to look at migrants’ participation in the 2012 electoral campaign. In particular, I focus on the interrelations between economic remittances and electoral behaviors. By doing so, I show how migrants’ economic resources foster their political influence on non-migrants. More specifically, I take advantage of a series of questions aiming at measuring migrants’ attempts to influence the voting behaviors of their kin at origin by giving them *explicit voting recommendations* (see Table 14 for wording of the questions). A striking pattern emerging from the data is the high proportion of migrants who declared they strived to influence their kin at origin, as well as the conviction that their advice had been followed. Two thirds of the sample of migrants said they encouraged family members to vote and more than half of the sample (52 % in the U.S., 58 % in France) declared that their family members took the recommendation into account (Table 12).

**Table 12:** The impact of migrants’ voting recommendations -- according to migrants

Voting advice were followed (marginal effects)	1	2	3	4	5	6
Age (years)	0.000304	-0.000144	-0.000404	-0.000545	-0.00202	-0.00120
	(0.20)	(-0.09)	(-0.25)	(-0.35)	(-1.28)	(-0.77)
Sex (female)	-0.0642	-0.0844*	-0.0562	-0.00626	-0.0224	-0.0101
	(-1.57)	(-2.08)	(-1.35)	(-0.16)	(-0.55)	(-0.25)
Lenght of stay at destination	-0.0000343		-0.0000362	-0.0000415	-0.0000347	-0.0000402
(years)	(-1.07)		(-1.15)	(-1.43)	(-1.26)	(-1.45)
Diploma : High school	0.0463**		0.0399*	0.0209	0.0197	0.0151
(ref: none)	(2.97)		(2.57)	(1.40)	(1.25)	(1.02)
Diploma : Some college		0.0466	0.0395	-0.00911	0.00561	-0.0182
		(0.82)	(0.69)	(-0.17)	(0.10)	(-0.34)
Diploma: Bachelor		-0.0335	-0.0354	-0.0954	-0.0633	-0.0969
		(-0.61)	(-0.65)	(-1.80)	(-1.19)	(-1.83)
Annual income:$16,000 to			0.0804		0.0625	
$40,000			(1.41)		(1.13)	
Annual income			0.0311		0.0305	
More than $40,000			(0.45)		(0.45)	
Annual income: NR			-0.00658		0.0268	
Ref: less than $16,000			(-0.12)		(0.49)	
Talk about politics with				0.231***		0.209***
Natives (at destination)				(6.74)		(5.97)
Send economic				0.135**	0.131**	0.118**
Remittances (ref : no)				(3.02)	(2.80)	(2.64)
Plan to return to Senegal				0.0926*	0.0967*	0.0942*
(ref : no)				(2.34)	(2.42)	(2.39)
Member of a HomeTown					0.0646	
Association (HTA) (ref : no)					(1.69)	
Unionized at destination						0.0998*
(ref : no)						(2.22)
Member of a political party					0.155***	
At destination (ref : no)					(4.42)	
Participation in the elections						0.0681
At destination (ref : no)						(1.83)
N	754	754	754	754	754	754

Note: Table reports average marginal effects of the logit specification. The marginal effects are evaluated at the means of the data. For binary variables, the marginal effects are the differences between the two categories of the independent variable. All regressions include country-fixed effects. T-statistics in parentheses (*p<0.1 ** *p*<0.05, *** *p*<0.01). Source: SSU survey (DIAL, IRD 2012)

Under which conditions do migrants think that they influenced their relatives back home? The multivariate models (see Table 12) show that more educated migrants, with more experience of the political sphere of their host country tend to think they are more influential. Above all, these results confirm the channels through which social remittances circulate: the link between being influential and sending monetary transfers is positive and statistically significant in the different estimations. The statistical correlations between the circulation of economic transfers and the circulation of voting recommendations corroborate the hypothesis that remittances have the power to reinforce asymmetrical relationships.

### Economic remittances and voting advices

It is necessary to be cautious at this stage because individuals’ propensity to declare influencing others might be subject to bias that would lead to an overestimation of the impact of migrants’ voting recommendations. To address this concern, I turn to the data collected in Senegal, with non-migrants. Despite the smaller size of the sample, the same correlations appear: non-migrants are more likely to listen to migrants’ voting recommendations if they receive economic remittances from the latter, *all other things held equal* (Table 13).

**Table 13:** The impact of migrants’ voting recommendations according to non-migrants

Followed Voting advice (non-migrants) (Marginal effects)	(1)	(2)	(3)	(4)	(5)
Age (years)	0.00272	0.00270	0.0198	0.0266	0.0105
	(1.16)	(0.88)	(1.18)	(1.55)	(0.58)
Sex (female)	0.0585	0.117	0.870	0.895	0.407
	(1.02)	(1.52)	(1.89)	(1.82)	(0.97)
Education: High School	0.101	0.0868	1.117*	1.118*	
(ref. none)	(1.60)	(0.83)	(2.38)	(2.47)	
Some College	0.0866	0.128	1.450*	1.628**	
	(1.19)	(1.23)	(2.24)	(2.77)	
Bachelor	0.125	0.147	1.613*	1.484**	
	(1.09)	(1.35)	(2.54)	(2.80)	
Receive Economic	0.121**	0.168*	1.436**	1.548**	1.249*
Remittances	(2.79)	(2.12)	(2.63)	(2.96)	(2.55)
Talk politics with natives		0.214***	1.600***	1.770***	1.537***
(ref : no)		(3.53)	(3.81)	(3.99)	(3.54)
Freq. of Phone		-0.00405	0.363	0.405	0.354
Calls : weekly		(-0.06)	(1.17)	(1.28)	(1.03)
(ref: daily)					
Monthly		0.118	0.945	1.240*	0.406
		(1.07)	(1.57)	(2.17)	(0.64)
Never		0.265	1.202	1.088	0.878
		(1.82)	(1.64)	(1.52)	(1.44)
Freq. Visits			-0.625	-0.725	-0.825*
To Senegal (<yearly)			(-1.47)	(-1.66)	(-2.16)
(ref: > yearly)					
Less			0.168	0.00140	-0.0986
			(0.42)	(0.00)	(-0.27)
Never			0.996	1.285	0.670
			(1.25)	(1.61)	(0.97)
Employed				0.460	
(ref. unemployed)				(0.79)	
Unionized (yes)					-0.390
(Ref. no)					(-0.70)
N	163	163	163	163	163

*Note*: Table reports average marginal effects of the logit specification. The marginal effects are evaluated at the means of the data. For binary variables, the marginal effects are the differences between the two categories of the independent variable. All regressions include country-fixed effects. T-statistics in parentheses (*p<0.1 ** *p*<0.05, *** *p*<0.01). Source: SSU survey (DIAL, IRD 2012)

These results support the claim that economic *“remittances are just one mechanism”* through which the diaspora has a political impact on the country of origin*. “Members of diasporas participate in the politics of their country of origin in a variety of ways*”. In particular, expatriates “*influence the voting preferences of kin in the country of origin, an influence that is amplified if they send financial remittances”* (Kapur, 2010, 2014, p. 484). This paper gives empirical support to such claims and it also illuminates: *why* financial remittances foster migrants’ political influence.

A theoretical approach to economic sociology can encompass and further these conclusions by showing *why* economic transactions foster intimate but asymmetrical ties. Indeed, “*with its focus on the interactional efforts at negotiating economic relations, infused with sense-making, that have implications for power distribution between partners to an exchange, the concept of relational work is poised to serve economic sociology”* (Bandelj, 2012) and migration studies.

### Why?

Economic remittances affect the hierarchy between the sender and the recipient. Hence, the correlations between receiving economic remittances and following voting recommendations cannot be reduced to mere clientelistic relationships; the reality is more complex. To be sure “*Money matters in politics […] But other things matter as well, and the direct correlation between money and outcomes that so many political scientists have sought simply is not there”* (Baumgartner, 2009, p. 291). More precisely, the statistical correlations between economic transfers and political influence signal complex interdependencies that cannot be conflated to presence or absence of clienteslistic patronage. Complex practices renegotiate, reshape, nuance, and rework the role of economic resources when it comes to political mobilization. Non-migrants have their own agency: in polling booths, they might ignore migrants’ recommendations and cast their ballot for another candidate. However, fieldwork showed that non-migrants are susceptible of aligning their political preferences on the ones of migrants, for a series of reasons.

In countries where emigrants provide the population with financial contributions that significantly affect living conditions, non-migrants might have real incentives to vote for a candidate recommended by migrants. Non-migrants might think their personal interest coincides with that of migrants: given Senegal’s dependency on the resources of migration, it makes sense for the former to think a government that would protect migrants’ interests, or encourage the diaspora to invest in the country, will also (more or less directly) serve their own interest.

Economic dependency favors a political alignment. The following quote, from Issa Keira (37 years old), corroborates this point. He left Senegal at age 17 and has been working in Marseille (South of France) for more than 20 years at the time of this interview. His two wives and nine children almost exclusively rely on his remittances for their daily needs. He sends money at least once a month and visits Senegal at least once a year. In 2012, he travelled twice to Senegal, notably to support Macky Sall’s candidacy. He was actively involved in the 2012 electoral campaign. When I asked him about his participation, he replied:

*“What do you mean? Financially or physically?*

*- Financially.*

*- Sure. Well (…) in Europe or in Africa, you know, campaigns are not the same. Here in Africa, you have to give [money] (…) you have to make them [the voters] understand. Because, here, they rely on migrants [émigrés] for their living. Politics… they don’t care about politics.*

*- They don’t care?*

*- What do they even know about it? The main thing for them is: getting enough to eat. I have to make them understand that if they wanna keep eating, they have to come vote for us [for Macky Sall, the candidate he is supporting]”*

Interview with Issa Koïta,

Dakar (Senegal). April, 19^th^, 2012.

When one person, like Issa, provides the living of a dozen individuals back home, this breadwinner is likely to exert a real influence on those who stayed behind. Unequal exchanges, such as remittance transactions, *“contribute to and reinforce honor, prestige, and authority”* of one actor on the other, Eckstein points out (2010, p. 1651). Consequently, remittances might be reciprocated through recognition of the sender’s moral virtue and *“concomitant support of their social standing*” (Åkesson, 2009). The fact that non-migrants take into consideration migrants’ advice can be seen as a form of support of their social role in their community and country of origin.

More broadly, migrants can leverage their economic resources to foster their political influence. Diasporas “*exhibit more active persuasion stemming from a didactic role in stimulating the international transmission of ideas*”, notably because they are more likely to be economically successful than those remaining at home (Kapur & McHale, 2005, p. 122). The following quote, from Chillo Bomou, illustrates this point. In this excerpt, this 39-year old Senegalese woman living in a village hailed with high emigration rates, explains why she supports Macky Sall’s candidacy (in 2012), after supporting Abdoulaye Wade (the outgoing president) for more than 10 years:

“*Who told you about Macky [Sall]?*

*- My husband convinced me [to vote for Macky Sall]*

*- Who told about Macky [Sall] to your husband?*

*- Syabou, Issa [Keira, both current migrants living in France]*

*- And you listened to them?*

*- Sure. They are good friends. Every time they are coming back from France, they offer us gifts (…) clothes, lots of clothes. And money too. (…) they are good friends.*

*- But… I imagine that you have other friends. All of them do not vote for Macky Sall?*

*- No.*

*- So how did you decide to follow these friends rather than other friends?*

*- Well. It’s a matter of personal interest too. They are our neighbors (…) they offer us many gifts. So, if they told us this [to vote for this candidate], (…) they bring back many things, to the village as well. We want them to keep doing this. So we go where our interest is, too.”*

Interview with Chillo Boumou,

Diarama^25^ (Tambacounda), Feb. 26th, 2012

Beyond the relational argument, Chillo puts forth a plausible mechanism that empowers migrants. Indeed, the fact that migrants’ resources loom large in the Senegalese economy also explains migrants’ voice in Senegalese politics. The prestige associated with migrating (especially in countries such as France or the U.S.), the economic resources, and the social capital that migrants acquire in exile participate in their enhanced ability to be listened to. This is all the more true in a country such as Senegal, where common discourses depict emigrants as a symbol of success or “gold mines;” the latter being preferred by women and mothers as potential husbands (Riccio, 2005, p. 107) and admired by the young as role models (Fall et al., 2010). Poised with increased economic resources and prestige, migrants who maintain lively transnational ties are also likely to be more influential when it comes to politics.

## Fifth section: conclusion

The concept of social remittances opened a new field for migration studies and triggered a welcomed shift in research focus from the economic to the sociopolitical implications of human mobility. However, the fast growing scholarship on social remittances, in a legitimate attempt to place the social at the center of research agendas, might have gone too far in conceptualizing economic remittances and social remittances as distinct, if not antagonist, flows. As a result, this body of literature disregards the extent to which migrants’ influence is fostered by their material resources.

Through the analysis of the 2012 Senegalese presidential election, this paper makes the case that economic remittances bolster the circulation of social remittances. It combines three sources of empirical evidence – (1) census data, (2) survey data, and (3) qualitative data – to compare the transnational practices of Senegalese-born migrants located in the U.S. and in France. This comparative lens discloses that, despite contrasted trajectories at destination, Senegalese migrants exhibit a high propensity to engage in transnational political and economic practices, and notably to remit money back home. These economic transfers contribute to the maintenance of transnational interpersonal ties. In other words, economic remittances perform a transnational *relational work* (Zelizer, 2005, 2012): these economic transactions reaffirm and strengthen relationships, in spite of the distance. These transnational relationships are inherently asymmetrical. In the case of Senegal, non-migrants’ dependence on expatriates’ financial support enhance migrants’ propensity to influence their kin. Migrants’ economic power increases their voice, and their ability to significantly affect electoral outcomes in their home country. Senegalese-born migrants settled in the U.S. and in France are endowed with material resources that foster their political influence.

Several questions remain for further research. A line of inquiry could further compare the trajectories of African-born migrants located on both sides of the Atlantic in order to investigate the existence of “reverse social remittances”. Indeed, the intuition that social remittances are not unidirectional but *“circulate, continuously and iteratively”* (Levitt & Lamba-Nieves, 2011) calls for further empirical evidence. Further research is also needed to better understand the role of transnational economic resources in national politics. To start with, more empirical data would be necessary to investigate how migrants fund political parties and campaigns, and more broadly, to compare the extent to which the money of migrants differ (or not) from other transnational financial flows.

In this respect, economic sociology opens up promising areas for research by moving forward our understanding of the social and cultural dimension of monetary transactions. It paves the way to unpack the material dynamics underpinning the circulation of ideas and practices. This project thus invites scholars to push the boundaries of migration studies in order to engage in interdisciplinary cross-talk with researchers exploring distinct empirical questions but related theoretical issues.

**Table 14:** Variables of interest

Independent variables	Wording	Nature
Age (years)	Age (in years)	Continuous
Sex (female)	Sex	Binary
Length of stay abroad	Date of arrival in France/the U.S.	Continuous
Occupation	Current profession?	Discrete (cf. Table 1)
Citizenship (single or dual)	Current citizenship	Discrete (cf. Table 1)
Income	Total salary from regular job	Discrete (cf. Table 1)
Educational attainment	Highest degree obtained	Discrete (cf. Table 1)
Dependent variables		
Frequency of phone calls	How often do you call your relatives in Senegal?	Discrete
Frequency of visits	How often do you usually visit your relatives in Senegal?	Discrete
Send economic remittances	Do you send money to your country of origin?	Binary
Own a land/house in Senegal	Do you own a plot of land in Senegal, a house, or an apartment, even in it is still in construction?	Binary
Own a business in Senegal	Do you own a business or are you personally committed to any business venture in Senegal?	Binary
Plan to return to live in Senegal	Do you plan to go back home definitively?	Binary
Vote in the country of destination	Did you vote in any French/US election?	Binary
Vote in Senegal	Did you vote: - In the 2000 Senegalese presidential election? - In the 2007 Senegalese presidential election?	Binary (1 if the respondent said “yes” to at least one item)
Campaigned in 2012	Have you been personally active in the (2012) Senegalese presidential campaign?	Binary
Member of a political party in Senegal	Are you/have you ever been member of a any political party in Senegal?	Binary
Member of a political party at destination	Are you/have you ever been member of a any political party in France/the U.S.?	Binary
Voting advice given by migrants were followed (according to migrants)	Did you encourage your family members to vote? Did they take into account your opinion?	Binary (1 if the respondent replied “yes” to both questions; 0 otherwise)
Voting advice given by migrants were followed (according to non-migrants)	Did [this migrant] encourage you to vote for a given candidate? Did you take into account her/his opinion?	Binary (1 if the respondent replied “yes” to both questions; 0 otherwise)

Source: SSU survey (American, French, and Senegalese questionnaires). DIAL, IRD (2012)

## Footnotes

1 This figures the votes cast in Senegalese consulates in the U.S. and in France, in the second round of the 2012 Senegalese presidential elections. Source: Commission Eléctorale Nationale Autonome (CENA).

2 Source : Jeune Afrique, March 12, 2012. (http://www.jeuneafrique.com/177017/politique/s-n-gal-la-coalition-de-macky-sall-se-renforce-abdoulaye-wade-cherche-l-appui-des-religieux).

3 Their indicators reflect the six following categories: “Voice and Accountability (VA), Political Stability (PS), Government Effectiveness (GE), Regulatory Quality (RQ), Rule of Law (RL) and Control of Corruption (CC) covering about 200 countries and territories for five years: 1996, 1998, 2000, 2002 and 2004” (Li & McHale, 2006, p. 10).

4 Census data, cf. third section.

5 Cf. the report edited by the African Development Bank http://www.afdb.org/fileadmin/uploads/afdb/Documents/Publications/70000012_EN_Transfert%20des%20fonds%20ANG.pdf (retrieved May 3, 2014).

6 Organisation internationale pour les migrations, Migration au Sénégal, Profil National 2009, citing ANSD 2001 statistics published in 2004.

7 Of the Senegalese Democratic Party (Parti Democratique Sénégalais or PDS).

8 Cf. http://www.lemonde.fr/afrique/article/2012/03/26/au-senegal-abdoulaye-wade-reconnait-sa-defaite_1675626_3212.html

http://www.nytimes.com/2012/03/26/world/africa/president-concedes-race-in-senegal.html.

9 « Le président Wade explique à qui veut l’entendre que s’il n’a pas gagné au premier tour, c’est parce que « l’Occident a fait campagne électorale contre » lui, sans donner plus de détail mais en visant vraisemblablement au moins la France et les États-Unis ». Source : Jeune Afrique, March 12, 2012. Source: http://www.jeuneafrique.com/177017/politique/s-n-gal-la-coalition-de-macky-sall-se-renforce-abdoulaye-wade-cherche-l-appui-des-religieux.

10 Source: Commission Electorale Nationale Autonome (CENA). See also Dedieu et al., 2014 for similar findings regarding the 2000 election.

11 Source: Commission Electorale Nationale Autonome (CENA). See also Dedieu et al., 2014 for similar findings regarding the 2000 election.

12 The TIMME project (Terrains Interdisciplinaires et Multi-Sites: Migrants et Engagements). Cf. website.

13 The MIDDAS project (Migrations Internationales et Développement: Données Appariées migrants-familles d’origine au Sénégal). Cf. http://www.dial.ird.fr/projets-de-recherche/projets-anr/middas

14 More details on ongoing projects here: http://www.dial.ird.fr/. All the data analyzed in this paper were collected by DIAL’s research team. The possibility to develop interdisciplinary surveys was critical to this work and was made possible by Flore Gubert and Sandrine Mesplé-Somps.

15 Questions T5 to T8 in the questionnaire (available upon request). See also the Table 14 (annexes) for wording of the questions.

16 See Table 14 (annexes) for wording of the questions.

17 Cf*.* https://www.census.gov.

18 In this paper, all the figures concern raw effectives.

19 Institut National d'Etudes Démographiques (INED)

20 Institut National de la Statistique et des Etudes Economiques (INSEE)

21 the sample covers all regions of metropolitan France but excluding overseas territories

22 In this paper, all the figures concern weighted effectives.

23 The survey data (SSU) confirms these patterns: 55.2 % of those who are not planning on returning to Senegal declared that they were interested in the last presidential campaign; this proportion reaching 88.6 percent among migrants who are planning on going back to Senegal. The latter were also more likely to be members of a Senegalese political party, and to be actively involved in the campaign.

24 Source: Oxford dictionary (http://www.oxforddictionaries.com/us/definition/american_english/investment).

25 The names of persons and places were changed to protect the privacy of the interviewees.
